# Cancer predisposing syndromes in childhood and adolescence pose several challenges necessitating interdisciplinary care in dedicated programs

**DOI:** 10.3389/fped.2024.1410061

**Published:** 2024-06-03

**Authors:** Stefanie Kaffai, Daniela Angelova-Toshkin, Andreas B. Weins, Sonja Ickinger, Verena Steinke-Lange, Kurt Vollert, Michael C. Frühwald, Michaela Kuhlen

**Affiliations:** ^1^ Pediatrics and Adolescent Medicine, Faculty of Medicine, University of Augsburg, Augsburg, Germany; ^2^ Augsburger Zentrum für Seltene Erkrankungen, University of Augsburg, Augsburg, Germany; ^3^ MGZ-Medizinisch Genetisches Zentrum, Munich, Germany; ^4^ Department of Diagnostic and Interventional Radiology, University of Augsburg, Augsburg, Germany

**Keywords:** predisposition, children, surveillance, preventive testing, cancer, hereditary

## Abstract

**Introduction:**

Genetic disposition is a major etiologic factor in childhood cancer. More than 100 cancer predisposing syndromes (CPS) are known. Surveillance protocols seek to mitigate morbidity and mortality. To implement recommendations in patient care and to ascertain that the constant gain of knowledge forces its way into practice specific pediatric CPS programs were established.

**Patients and methods:**

We retrospectively analyzed data on children, adolescents, and young adults referred to our pediatric CPS program between October 1, 2021, and March 31, 2023. Follow-up ended on December 31, 2023.

**Results:**

We identified 67 patients (30 male, 36 female, 1 non-binary, median age 9.5 years). Thirty-five patients were referred for CPS surveillance, 32 for features suspicious of a CPS including café-au-lait macules (*n* = 10), overgrowth (*n* = 9), other specific symptoms (*n* = 4), cancer suspicious of a CPS (*n* = 6), and rare neoplasms (*n* = 3). CPS was confirmed by clinical criteria in 6 patients and genetic testing in 7 (of 13). In addition, 6 clinically unaffected at-risk relatives were identified carrying a cancer predisposing pathogenic variant. A total of 48 patients were eventually diagnosed with CPS, surveillance recommendations were on record for 45. Of those, 8 patients did not keep their appointments for various reasons. Surveillance revealed neoplasms (*n* = 2) and metachronous tumors (*n* = 4) by clinical (*n* = 2), radiological examination (*n* = 2), and endoscopy (*n* = 2). Psychosocial counselling was utilized by 16 (of 45; 35.6%) families.

**Conclusions:**

The diverse pediatric CPSs pose several challenges necessitating interdisciplinary care in specified CPS programs. To ultimately improve outcome including psychosocial well-being joint clinical and research efforts are necessary.

## 1 Introduction

Genetic disposition is a major etiologic factor in childhood cancer (1–6). Over recent years, there has been increasing awareness and recognition of childhood cancer predisposition syndromes (CPSs) (7). Considerable research efforts have led to substantial progress in the identification of CPSs in children affected by cancer (8–11). Tailored interventions implemented by rational surveillance protocols seek to mitigate morbidity and mortality through early detection and less toxic therapies (12). Most recommendations are based on history and physical examination combined with imaging and biochemical/metabolic studies. In some instances, whole body magnetic resonance imaging is indicated, e.g., in the case of Li-Fraumeni syndrome (LFS) or rhabdoid tumor predisposition syndrome (13–15).

Incidental findings resulting from those studies may lead to additional and potentially rather invasive diagnostic tests (16, 17). Both the anticipation of a particular test and the waiting time to results elicit anxiety (also referred to as “scanxiety”) (18, 19). The phenomenon of scanxiety, however, remains understudied. In addition, specialized centers such as CPS centers, pediatric oncology clinics, and institutes of human genetics are severely under-resourced in psychology support.

Individuals with CPSs carry a significantly increased risk of developing one or more cancers in a metachronous fashion. In 2016, the American Association of Cancer Research sponsored a pediatric cancer predisposition workshop to develop international consensus recommendations for cancer surveillance of children and adolescents for the 50 most common CPSs (12, 20). The multi-disciplinary working group decided that surveillance should be recommended when there is a ≥ 5% risk of developing cancer during the first 20 years of life and when effective screening modalities are available (12). Surveillance recommendations focused on the type(s) of cancer to which the individual is most likely predisposed and the time of greatest risk. For conditions with a cancer risk between 1%–5% surveillance may be indicated on an individual basis.

As more than 100 childhood CPSs are currently known, an abundance of CPS is on record for which no surveillance protocol exists. Many of those CPSs are (very) rare complex diseases with severe coexisting conditions. Information regarding cancer predisposition is not always in place and used to guide management (11).

We previously reported a variety of rare diseases included in the spectrum of CPSs in a tertiary-care children's hospital (21). Given the clinical relevance of a CPS in a child (e.g., surveillance, prevention, treatment, psychologic support, counselling, and identification of relatives at risk), awareness of cancer risk needs to be enhanced (22).

In order to improve tumor surveillance and, thus, care of children and adolescents with CPSs we established a dedicated pediatric CPS program. We here report our initial experiences asking which patients were referred, by whom, and for what reason. We further analyzed CPS diagnoses, surveillance recommendations, adherence to guidelines, and (psychosocial) challenges.

## 2 Patients and methods

We included all children, adolescents, and young adults referred to our pediatric CPS program at the Swabian Children's Cancer Centre with a proven or suspected CPS between October 1, 2021, and March 31, 2023. Follow-up for this study was completed on 31 December 2023. No exclusion criterion was defined. The study was performed in accordance with the Declaration of Helsinki and Good Clinical Practice and was approved by the ethics committee of the Ludwig Maximilian University of Munich (IRB number 23-0463), Germany.

Patients, parents, and physicians, respectively, were informed about the CPS program by flyers, information sessions, and personal contact. Information in layman's terms was provided on the hospital's homepage. A podcast and a television report about CPS including the program were promoted by the Bavarian Cancer Research Center (BZKF). Via the Augsburg Center for Rare Diseases (AZeSe), self-help groups were informed about the program.

Surveillance included clinical examination and anthropometric measurements in all patients complemented by neurological examination in patients at brain tumor risk. Abdominal ultrasound was performed trimonthly in patients at risk of Wilms tumor, hepatoblastoma, adrenocortical carcinoma, and pheochromocytoma/paraganglioma, neck ultrasound in patients at risk of thyroid carcinoma or paraganglioma. Whole body magnetic resonance imaging (MRI) was performed annually in patients at risk of sarcoma and once in patients with Neurofibromatosis type 1 (NF1) in case the patient did not need anesthesia, brain MRI in patients at risk of brain tumors, and abdominal MRI in patients at risk of pheochromocytoma/paraganglioma. Surveillance included x-ray of the lungs in patients with DICER1 syndrome, and biochemical testing (metanephrines and methoxytyramine) in patients at risk of pheochromocytoma/paraganglioma. No routine measurement of alpha fetoprotein was conducted. Patients with predisposition to leukemia or myelodysplastic syndrome had a complete blood count with manual differential trimonthly and bone marrow evaluation as appropriate. Upper gastroduodenal endoscopy and/or colonoscopy were performed in patients with gastrointestinal cancer syndromes. Biannual ophthalmologic examination was recommended in patients with NF1. Investigations depended on the patient´s age, if applicable the youngest age at cancer onset in affected family members, the underlying pathogenic variant (e.g., brain MRI in Gorlin-Goltz syndrome), and previous findings. The intervals and diagnostics varied age- and CPS-dependent. We reviewed medical reports to assess demographic data, presenting features, patient characteristics, family history, circumstances of referral, suspected diagnosis, diagnostics, recommendations, psychosocial support, and follow-up. Data were retrospectively categorized for referring (sub)specialities, referral reasons including leading presenting features, and circumstances of CPS diagnosis.

## 3 Results

A total of 67 children and adolescents with suspected or proven CPS were identified with a mean age of 8.4 years [standard deviation, 6.1 years] at initial presentation. Gender ratio showed a small female predominance [36 (53.7%) female, 30 (44.8%) male, 1 (1.5%) non-binary]. As of December 31, 2023, one patient with Noonan syndrome had died of respiratory failure as part of viral infection. Details on demographic data and presenting features at initial presentation are given in Table 1.

**Table 1:** Demographic details and presenting features on 67 children and adolescents referred to the specified paediatric CPS program.

Characteristics	CPS evaluation		CPS surveillance		Total	%
Patients	32	47.8	35	52.2	67	100
Gender						
Male	14	43.8	16	45.7	30	44.8
Female	18	56.3	18	51.4	36	53.7
Non-binary	0		1	2.9	1	1.5
Age at initial presentation, years						
Median, range	4.3 (0.2–19.5)		9.5 (1.5–20.5)		7.7 (0.2–20.5)	
Mean, standard deviation	7.0 (6.7)		9.9 (5.4)		8.5 (6.2)	
Presenting features						
CALM	10	31.3			10	14.9
Features of overgrowth^a^	9	28.1			9	13.4
Other specific symptoms	4	12.5			4	6.0
Cancer suggesting CPS^b^	4*	12.5*			4*	6.0*
Metachronous malignancies	1*	3.1*			1*	1.5*
Familial childhood cancers	2	6.3			2	3.0
Rare neoplasm^c^	3	9.4			3	4.5
First cancer due to CPS^d^			14	40.0	14	20.9
Predictive genetic testing			8	22.9	8	11.9
Syndrome with cancer predisposition			12	34.3	12	17.9
CPS incidentally diagnose			1	2.9	1	1.5
CPS, clinically diagnosed						
No	3	9.4	0	0	3	4.5
Yes	6	18.8	21°	60.0	27	40.3
Not applicable	23	71.9	14	40.0	37	55.2
CPS, genetically confirmed						
No	6	18.8	1^+^	2.9	7	10.4
Yes	8^#^	25.0	32	91.4	40	59.7
Not tested	18	56.3	2	5.7	20	29.9
Familial CPS carrier, subsequently identified						
Parents	3	9.4	1	2.9	4	6.0
Siblings	1	3.1	1	2.9	2	3.0
Not tested	1	3.1	1	2.9	2	3.0
Neoplasm detected by surveillance						
First neoplasm	0	0	2	5.7	2	3.0
Metachronous tumour	1	3.1	3	8.6	4	6.0
Deceased as of December 31, 2023	0	0	1	2.9	1	1.5

^a^ Features of overgrowth included macroglossia, hemihyperplasia, omphalocele, neonatal hypoglycemia/prolonged hyperinsulinism, macrosomia, embryonal tumors, visceromegaly, adrenocortical cytomegaly, kidney abnormalities, ear creases/posterior helical ear pits (23).

^b^ Pheochromocytoma, paraganglioma, medullary thyroid carcinoma, oncocytic (thyroid) adenoma DD thyroid carcinoma.

^c^ Malignant melanoma of the conjunctiva, acinic cell adenocarcinoma of the parotid gland, nodular sclerosing adenosis of the breast.

^d^ Osteosarcoma (*n* = 1), retinoblastoma (*n* = 3), hepatoblastoma (*n* = 1), myelodysplastic syndrome (*n* = 4), nephroblastoma (*n* = 1), Sertoli Leydig cell tumor (*n* = 1), leukemia (*n* = 1), optic pathway glioma (*n* = 2).

* With metachronous bilateral pheochromocytoma.

^#^ With Kabuki syndrome.

° Clinically suspected and confirmed by genetic testing.

^+^ Fanconi anemia clinically suspected, functionally confirmed, no pathogenic variant identified.

**Figure 1:**
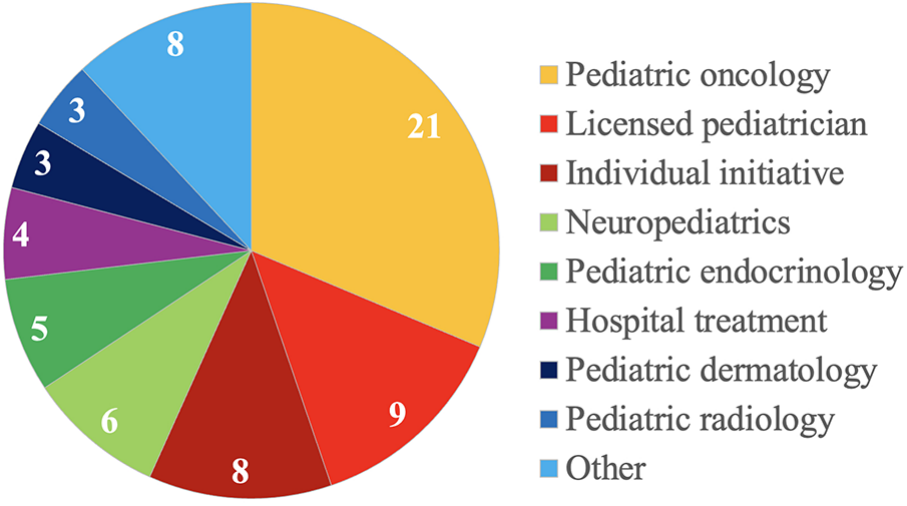
Details on referring specialties of 67 children and adolescents with suspected and proven cancer predisposition syndromes.

Of 67 patients, 21 (31.3%) patients were referred by pediatric oncologists, 9 (13.4%) patients by licensed pediatricians, and 8 (11.9%) patients presented on their individual initiative. Details on referring (sub)specialities are depicted in Figure 1.

**Figure 2:**
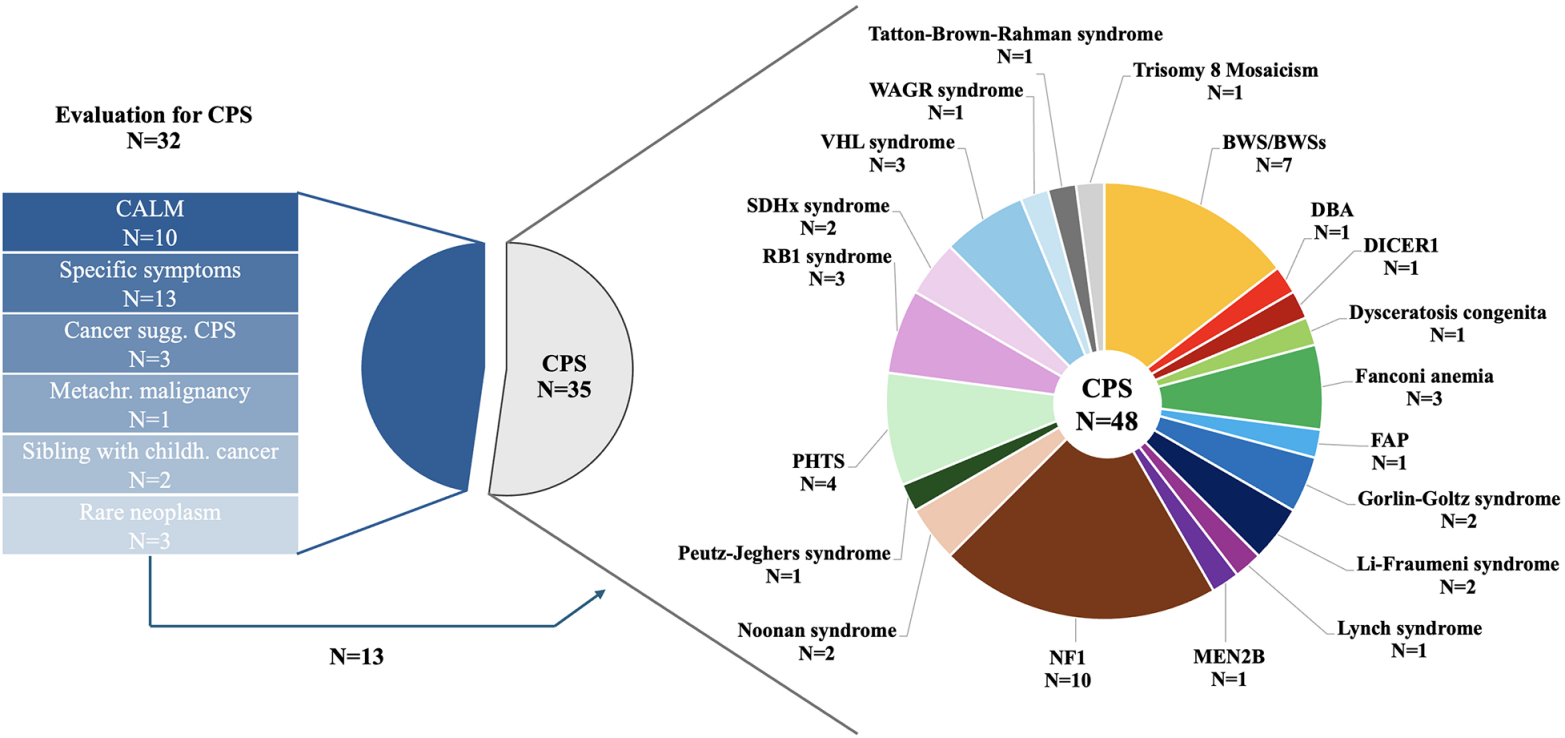
Reasons for referral in 67 children and adolescents presenting to the CPS program and details on CPS diagnosis in 48 patients.

In total, 35 (52.2%) patients presented for CPS surveillance, 32 (47.8%) patients for evaluation of an underlying CPS (Figure 2). Of those patients referred for CPS evaluation, 10 (31.3%) patients presented with café-au-lait macules (CALM), 9 (28.1%) patients with suspicion of an overgrowth syndrome, 4 (12.5%) patients with other specific symptoms/congenital anomalies, 4 (12.5%) patients with a childhood cancer highly correlated with specific genetic syndromes and/or molecular analysis suggesting CPS including 1 (3.1%) patient with two metachronous malignancies, 2 (6.2%) siblings with cancer <18 years of age, and 3 (9. 4%) patients with rare neoplasms.

Genetic testing was initiated in 5 of 10 patients with CALM, 3 of 13 with other specific symptoms, 4 of 6 patients with high suspicion of an underlying CPS, both siblings, and none of the 3 patients with rare neoplasms. Genetic testing confirmed CPS diagnosis in 7 (of 13; 53.8%) of those patients and Kabuki syndrome in one patient.

Of 10 patients with CALM, the diagnostic criteria ≥6 CALM >5 mm in greatest diameter in prepubertal individuals and >15 mm in greatest diameter in postpubertal individuals was not fulfilled in 4 patients. One further patient presented with clinical diagnosis of segmental NF1. A total of 6 patients with suspicion of an overgrowth syndrome met diagnostic criteria, 3 patients did not show signs of an overgrowth syndrome.

Of 35 patients referred for CPS surveillance, 14 (40.0%) patients were diagnosed after first cancer onset, 8 (22.9%) patients had undergone predictive genetic testing because one parent carried a pathogenic variant (PV) in a cancer predisposing gene (CPG; *PTCH1* *n* = 2, *VHL* *n* = 2, *SDHC* *n* = 1, *PTEN* *n* = 3) with risk of cancer onset during childhood, 12 (34.3%) patients were referred for an underlying syndrome with increased cancer risk, and 1 (2.9%) patient was incidentally diagnosed with trisomy 8 mosaicism.

In total, 6 other so far unaffected relatives carrying the CPG PV were subsequently identified in LFS (parent, adult sibling), Lynch syndrome (parent), DICER1 syndrome (parent), and PTEN hamartoma tumor syndrome (PHTS; parent, sibling). One parent was subsequently diagnosed with cancer. In two other patients (LFS, PHTS), parents refused genetic testing of themselves. These data highlight the importance of genetic counselling and testing.

Investigating these 48 patients with CPS in more detail, surveillance protocols were available for 45 (93.8%) including 2 with Noonan syndrome, for which surveillance is currently not recommended. Surveillance protocols were not in place for patients with pediatric Lynch syndrome, Tatton-Brown-Rahman syndrome (TBRS), and hematopoietic trisomy 8 mosaicism. The latter patient presented with thrombocytopenia and increased mean corpuscular volume and was therefore included in the CPS program. The parents of one patient with Noonan syndrome and TBRS explicitly wished to be included in the CPS program.

A total of 45 patients were eventually included into our dedicated CPS program. One patient with LFS did not present for follow-up due to parental refusal, 8 patients including 5 adolescents and young adults only sporadically kept their appointments.

Psychosocial counselling was offered to all patients included in the CPS program; 16 (35.6%) families made use of this support for psychological (*n* = 2), social (*n* = 9), and psychosocial (*n* = 5) reasons. Support was provided to parents (*n* = 12) and families (*n* = 5) at a median of 2.5 times (range, 1–20).

During the study period, surveillance revealed one first neoplasia by clinical suspicion (highly suspicious vision in clinical examination through fun and games in a toddler with NF1, subsequently confirmed as opticus glioma by MR imaging) and a further one by radiological examination (suspicion of thyroid carcinoma by neck ultrasound; PHTS). Three metachronous tumors were identified by radiological examination (*n* = 1; follicular-patterned thyroid tumor in DICER1 syndrome) and endoscopy (*n* = 2; colon cancer in Familial Adenomatous Polyposis, carcinoma *in situ* in Peutz-Jeghers syndrome). In addition, in one patient with PHTS, a mass in the foot was detected by clinical examination and subsequently confirmed as arteriovenous malformation by radiological examination and pathological evaluation.

## 4 Discussion

We report our experiences of a newly established program specifically dedicated towards children, adolescents, and their families affected by cancer predisposition at a tertiary care children's hospital in Germany over a 1.5-years-period. Of 67 children and adolescents, 48 (71.6%) patients were diagnosed with a CPS, whereas the diagnosis of a CPS was not confirmed in 19 (28.4%) patients.

As might be expected, most patients were referred by pediatric oncologists (21, 24). However, 13.4% of patients were referred by licensed pediatricians, 11.9% presented on the parents’ initiative. It remains difficult to calculate numbers of children and adolescents affected by one of the various CPS and, thus, to estimate “real numbers” of children and adolescents living with a CPS in our catchment area (24). To raise awareness for children and adolescents with CPS, we provided information on our CPS program at various levels (21). And indeed, most licensed pediatricians and in-house pediatric subspecialists referred patients for that reason. It must be assumed, that there is still a significant number of patients who do not present to our CPS program yet.

Evaluation for an underlying CPS was mostly initiated for CALM and suspicion of overgrowth. Diagnostic criteria of NF1 were established by the National Institute of Health (NIH) in 1987 and revised in 2021 by Legius et al. (25). The NIH criteria relied on clinical features mainly not being present in early infancy although surveillance should be initiated at birth and diagnosis of NF1, respectively. In addition, opticus glioma manifest at a median age of 5 years whereas most diagnostic criteria only later in life (26). We previously demonstrated earlier diagnosis of NF1 by applying the revised diagnostic criteria, in particular by performing genetic testing in infants not fulfilling diagnostic criteria hitherto (27). We thus initiated genetic testing in 5 infants fulfilling the criterion `CALM´ as defined previously (25) or presenting with otherwise suspicious CALM, e.g., constitutional mismatch repair deficiency (28), and arranged re-evaluation in some young patients presenting with CALM not yet meeting the diagnostic criterion of NF1.

Overgrowth syndromes are associated with cancer onset in infancy (29–32). Thus, starting surveillance as early as possible is of crucial importance (29, 33). Recommendations for surveillance in the several overgrowth syndromes vary substantially including advice for genetic testing (33–35). We decided upon inclusion in our CPS program on clinical criteria and performed genetic testing on an individual basis after careful discussion with both caregivers.

Of 48 patients with CPS, this was only diagnosed after first cancer onset in 40% of patients including three patients with otherwise typical signs and symptoms (Multiple Endocrine Neoplasia type 2B, PHTS, Fanconi anemia) (36–38). In addition, in both PHTS families other family members presented with symptoms of PHTS before (37). This once more highlights that more awareness to and knowledge about the various CPS is still needed among pediatricians and other experts (21).

Of 8 patients diagnosed by predictive testing, 2 patients each with von Hippel-Lindau (VHL) syndrome and Gorlin-Goltz syndrome had previously been cared for by non-CPS specialists. The siblings with Gorlin-Goltz syndrome due to a PV in *PTCH1* underwent sequential cranial MR imaging in anesthesia though this is recommended for children with PVs in *SUFU* only (39, 40). In the siblings with VHL syndrome, biochemical testing had comprised catecholamines instead of metanephrines and 3-methoxytyramine (41–43). This impressively illustrates the need for specialized CPS care.

By referring at-risk relatives for genetic testing, we additionally identified 6 so far unaffected family members. One parent subsequently felt ill with cancer underlining the importance of secondary genetic testing of at-risk relatives (17, 44).

Management strategies encompass regular clinical examination, biochemical testing, radiological examination including MR imaging, and endoscopy for early detection of neoplasms (14, 15, 33, 41, 45–50). This requires both extensive human and instrument-based resources. However, lack of insurance coverage limits its availability. By clinical and radiological assessment, we identified 6 patients with new neoplastic manifestations during the study period. This highlights the potential of regular surveillance (51, 52).

We identified 8 patients not regularly keeping their appointments for various reasons. To what extent the phenomenon of “scanxiety” may be accountable is beyond the scope of this study. However, coping strategies and specialized psychosocial support are essential to individuals carrying a cancer predisposing PV (53–55). On the one hand undergoing tailored surveillance for an underlying CPS can lead to increased feelings of control and security (56, 57). On the other hand, it may prompt uncertainties while waiting for the test results and cause worries to the reminder of cancer risk as well as the practical aspects of screening (57).

Beyond that, genetic testing itself represents a major life event while the implications of testing might not be fully understood at the time of decision-making (58, 59). In addition, individuals may experience distress or clinically significant anxiety, especially those who have lost a close relative to the CPS (60, 61).

Survey instruments for psychosocial aspects of pediatric CPS are not available. In addition, psychosocial support within the frame of dedicated pediatric CPS programs is not covered by health insurance funds in Germany to date. Considering the number of individuals in our study who made use of psychosocial counselling, did not keep their surveillance appointments, or refused genetic testing, there are several psychosocial shortcomings which urgently need to be addressed in the future.

## 5 Conclusions

Pediatric CPSs include several challenges and opportunities necessitating interdisciplinary care in dedicated CPS programs. The multifaceted features of CPSs manifesting in childhood and adolescence merit increased awareness to facilitate identifying patients and at-risk relatives as early as possible. To ultimately improve outcome and psychosocial well-being of affected families joint clinical and research efforts—accompanied by education programs for patients, relatives, and physicians—are necessary.

## Data Availability

The original contributions presented in the study are included in the article/Supplementary Material, further inquiries can be directed to the corresponding author.
